# The provision of urban green space and its accessibility: Spatial data effects in Brussels

**DOI:** 10.1371/journal.pone.0204684

**Published:** 2018-10-17

**Authors:** Marion Le Texier, Kerry Schiel, Geoffrey Caruso

**Affiliations:** 1 UMR CNRS 6266 IDEES, Mont-Saint-Aignan, France; 2 University Rouen Normandie, Department of Geography, Mont-Saint-Aignan, France; 3 Institute of Geography and Spatial Planning, University of Luxembourg, Esch-sur-Alzette, Luxembourg; 4 Luxembourg Institute of Socio-Economic Research, Esch-sur-Alzette, Luxembourg; University College London, UNITED KINGDOM

## Abstract

Urban green space (UGS) has many environmental and social benefits. UGS provision and access are increasingly considered in urban policies and must rely on data and indicators that can capture variations in the distribution of UGS within cities. There is no consensus about how UGS, and their provision and access, must be defined from different land use data types. Here we identify four spatial dimensions of UGS and critically examine how different data sources affect these dimensions and our understanding of their variation within a city region (Brussels). We compare UGS indicators measured from an imagery source (NDVI from Landsat), an official cadastre-based map, and the voluntary geographical information provided by OpenStreetMap (OSM). We compare aggregate values of provision and access to UGS as well as their spatial distribution along a centrality gradient and at neighbourhood scale. We find that there are strong differences in the value of indicators when using the different datasets, especially due to their ability to capture private and public green space. However we find that the interpretation of intra-urban spatial variations is not affected by changes in data source. Centrality in particular is a strong determinant of the relative values of UGS availability, fragmentation and accessibility, irrespective of datasets.

## Introduction

In the past three decades a significant amount of scientific studies in urban geography, planning and environmental disciplines have shown that the environmental, ecological and social benefits of urban green space (UGS) vary according to size, distance and accessibility([1, 2], for early references). Regulatory bodies, at different scales, have set up a range of policies on UGS provision and access to ensure the development of greener cities [3]. For instance, the European Environment Agency [4] recommends that people should have access to green space within 15 min walking distance (1.61 kilometers / 1 mile). Despite this, average access to UGS is not sufficient because their spatial distribution may result in significant bias towards certain locations and hence, social groups [5–8]. Many researchers now argue for the extension of investigations into how parks are spatially distributed relative to social needs and reveal who has access to different kinds of UGS [7, 9]. However, while research on UGS provision and access inequalities has been proliferating and a variety of dimensions have been explored, there is still no methodological consensus about how a UGS is defined from data or how to conceptualise and measure its provision and access [5, 7, 10–16]. In particular the effect of different data sources, the public or private nature of UGS, and centrality effects are intermingled and still understudied.

In this paper we propose four complementary measures of UGS provision and access, applied to three datasets for Brussels. Recently [17] analysed the vegetation cover and access to public green spaces for the same study area for two indicators computed at the scale of individual buildings and citizens (via dasymetric mapping). Comparatively, we intend here to widen the set of urban green indicators and focus on identifying how different input data sources may bias results at both the aggregated (neighbourhood, city region) and disaggregated (pixel) levels. Our methods stress the multidimensional nature of UGS provision and access, thereby contributing to clarifying UGS definitions from data and their impact on measuring potential spatial inequalities. We suggest that there are strong effects of changing data source with respect to UGS definition and provision because of how the public/private status is recorded. We also find data effects on accessibility measurements at neighbourhood scale but suggest that centrality effects are limited.

### Provision and access dimensions

The absence of a shared definition of what a UGS is represents an important barrier for the generalisation of empirical studies [18, 19]. There are many dimensions in the literature regarding the definition of UGS but we have identified four of them related to provision and access because they have spatial variability within city regions, likely impacting the benefits people derive from UGS. The following literature is not meant to be exhaustive but to provide a background to our work.

#### Availability

Some authors have introduced a size threshold to the definition of green areas as UGS. For instance, [13] excluded all UGS smaller than 2 ha in their study, considering them to be too small to support the physical activity of adults. [7] stresses that the size of UGS not only matters in terms of the impact on the level of use and the type of activities people perform in UGS, but also because it affects the ecological qualities of UGS: the larger it is, the more diverse its flora and fauna are likely to be. This would affect the health and quality of life of close-by population as, for instance, UGS with lots of trees and green vegetation would decrease temperature, pollution rates, etc. The size of UGS is also likely to moderate the level of appropriation by different individuals, increasing the diversity of human values associated to a particular UGS [20]. The green space ratio is the most commonly used metric to refer to the availability of UGS [21] within a neighbourhood. It consists of calculating the amount (number and/or acreage) of UGS within a city or its sub-parts to provide an aggregate (or per neighbourhood) picture of provision to a certain number of residents, i.e. potential users [5] as well as potential UGS congestion [22, 23].

#### Fragmentation

[24] argue that total availability is not sufficient because it does not allow for distinguishing between areas with many small UGS units from areas with one large UGS unit located at its corner, thus raising the fragmentation issue. Fragmentation is important since small UGS are more likely to be evenly spread across a city but provide fewer benefits [9]. Theory also suggests that fragmentation can result from residential preference for close-by green space and that enlarging the provision of green from everyone’s home, rather than as a unique belt for instance, can increase utility [25, 26].

#### Public-private ownership

The ownership status of UGS has been considered as a substantial aspect of equality of provision as private UGS (private yards and gardens) offer an opportunity to compensate the absence of public UGS by other opportunities of contact with nature and recreational activities ([7, 12, 27], for e.g.). One should also note that studies incorporating only parks listed by local authorities overlook the value of informal green spaces—defined as areas covered at least partly with non-remnant and spontaneous vegetation—for no particular reason other than public ownership and policy habits and virtues [28]. However both private and informal UGS play a critical role in supporting biodiversity and providing ecosystem services in urban areas and act as a (partial) recreational substitute for public green space [12, 18]. Conversely, using the presence of vegetation as the only criteria to define UGS, would overestimate provision and access (and likely underestimate inequality of access) to UGS as it would include smaller units than an analysis focusing on formal green space [12, 29]. Others studies ([27], for e.g.) point out the fuzziness of the private/public distinction when it comes to UGS with restricted access (green space belonging to a residential subdivision that does not allow one beneficiary to exclude others) and private gardens whose aesthetic and ecological values accrue to adjacent neighbours.

#### Accessibility

Accessibility indices have been defined to measure relative remoteness and proximity to UGS, thus complementing availability within a neighbourhood with more accessible alternatives, which may be located outside the considered spatial entity [5, 23, 30]. Network approaches have been preferred to simpler Euclidean distances as these overestimate general access [5, 13, 31–34]. Other advancements have been put forth including the consideration of crowding to parks through the use of the two-step floating catchment area (2SFCA) method and/or access via different modes of transportation [35, 36].

Surprisingly, to our knowledge, there is no systematic analysis of the four dimensions above against how central different residential locations are. Green space availability and accessibility have been framed and analysed under spatial equity concerns but it is not the case for fragmentation. Moreover, if internal spatial heterogeneity is treated rather than aggregate outcomes, how distance to the center affects this heterogeneity is rarely analysed while endogeneity is very likely between residential choice and green space provision [8]. If it is obvious from standard urban economics that the consumption of private land increases with distance to jobs and hence that private green is also likely to increase with distance, little is known on how public green spaces are located along a distance gradient within the border of an urban area. Distance may well be the primary determinant of how UGS provision and availability indicators vary in space. This needs to be assessed.

The ecological and recreation services provided by UGS not only depend on their location, scale and shape, but obviously also on their vegetation structure, soil characteristics, management, potential accessibility and local urban context [3, 13, 16, 18, 37]. For instance, UGS with more recreational infrastructure or facilities as open sports or children’s play areas are often found to be the most attractive [23, 38, 39]. These features however come with extra data assemblage needs and are rarely available in a consistent manner across cases. They also have a lower spatial dimension and therefore will not be considered in the remainder of this work.

### Data effects

Less studied is the effect of data source on availability measures, although it has been acknowledged that the selection of data is likely to reflect only specific types of UGS [13, 40, 41]. There are five notable recent studies in this field, namely [17, 18, 42–44]. [42] compare the quantity of green space derived from the European land cover dataset Coordination of Information on the Environment (CORINE) and from the British Ordnance Survey’s master map (OSMM). They analyse their separate association with measures of mortality and morbidity at census ward level for the cities of York, Exeter, Edinburgh and Glasgow. They find that indicators based on the CORINE land cover tend to detect lower levels of green space exposure as the dataset mainly depicts the largest UGS. Interestingly, this does not affect the measured associations with the risk of mortality, suggesting a size effect in the mechanisms by which UGS influence health. [43] compare the quality of fifty parks selected from southwest Sydney, Australia, assessed using remote methods (using Google Earth Pro) and direct observation. They conclude that a strong correlation exists between the quality scores obtained using the two methods, but find a 1 to 10 saving in research time in favour of the remote-assessment method. [18] compare land use percentage obtained with publicly available high-resolution aerial photography data (Google Earth in Brisbane; Microsoft Bing Maps in Sapporo), surveyed land use type in the field (visual estimation) and city supplied datasets. They find that informal UGS land use types are more sensitive to data selection than formal ones. [44] compare maps of urban forest cover derived from user-generated data (PhillyTreeMap) to the one obtained from the Pennsylvania Geospatial Data Clearinghouse (using remote sensing methods). Their results show effects of census block demographic profiles on the completeness of PhillyTreeMap coverage: population density, housing vacancy, median home value, and percentage of white residents have positive statistically significant effects. In the case of Brussels, [17] found important differences in the spatial distribution of the green cover derived from QuickBird satellite imagery and the public green space locations provided by the Brussels Environment agency. In some peripheral parts of the city, walking distances to public green are indeed long but residents benefit from an abundance of private vegetation. Conversely, inhabitants of the city core benefit from a good access to public green space, but lack of vegetation in the immediate surroundings of their home.

These last three studies also show an emerging trend in UGS studies to embrace the digital turn in spatial data production and replace traditional data provision by governmental agencies and cartographic centres by data brought about by the Internet and social media such as Google Earth [18, 43], Google Street View [17, 45], or Twitter [46]. To our knowledge, there are no papers dealing with the potential added value of voluntary geographic information [47] such as OpenStreetMap (OSM) for UGS provision studies apart from [44]. With approximately 1.85 million registered users and contributors in 2014 [48], the ubiquity of OSM dataset offers a great potential for case study cross-analyses and results comparisons.

This paper is in line with the recent data effects literature and that related to the spatial dimensions of UGS provision mentioned earlier. We propose a method to critically examine the extent to which the data sources affect the different dimensions of green space provision and access, and eventually, our capacity to generalise across cases the source of spatial inequalities in the benefits obtained from UGS [18, 41]. The proposed procedure is based on measures of urban green space location and characteristics derived from two classical types of data, Landsat imagery and official cadaster-based map, and the voluntary geographical information provided by OpenStreetMap (OSM). Landsat and OSM, being available in many places, should allow for generalisation and transfer while the cadaster-based map is supposed to reflect the kind of institutional information available at local scale with most accurate details about formal UGS. Although OSM data can sometimes be based on cadastral datasets, it seems not to be the case here, as there is only a 73% overlap of OSM urban green space with UrbIS data, while 85% of UrbIS urban green space data overlaps with OSM data, thus showing different origins. In addition to this, the urban green space is represented slightly differently in the two datasets, with UrbIS data capturing the footpaths while OSM does not.

## Study area and methods

### Study area

The region of Brussels is located at the centre of Belgium, has a population of just over a million people and is divided up into 19 communes. Population is distributed along concentric gradients following the historical patterns of city development: Medieval city core, Ninetheen century urban expansion and mid-twentieth century suburbs and opposing a dense North-West area to more disperse housing patterns in the South-East. UGS within the Brussels region include many small formal parks and larger ones easily identifiable, such as the Park of Brussels, the Cinquantenaire park, or the park around the Atomium, built for the World Fair in 1958. At the southern edge of the region are the Bois de la Cambre and Forêt de Soignes, a very popular forested area for outdoor recreation, which according to a recent survey, encourages numerous and longer access trips by the inhabitants of the region [8]. The layer describing the limits of the Brussels region has been downloaded from the Brussels Region Informatics Centre (BRIC) website.

### Methods

We use three datasets in this study and analyse the consistency of four green provision and access metrics when applied to each dataset. We contrast remote sensing imagery from Landsat, the Open Street Map (OSM) layer as a a crowd-sourced vector map, and an official cadaster-based map, here named UrbIS. We have chosen the first two because of their ubiquity and their different spatial nature and data format, i.e. respectively continuous/discrete and raster/vector. The third one provides a control and, though it is also a vector, it is again of a different kind given it is produced by an institutional body.

Provision of and access to UGS are examined with respect to the spatial distribution of the four indicators discussed earlier in the literature section, namely (i) availability, (ii) fragmentation, (iii) privatisation and (iv) accessibility.

The indicators are computed as follows:

(i) The *availability index* is measured by the share of land dedicated to urban green space per area, i.e. total UGS cover *A* divided by the reference surface.
(ii) The *fragmentation index* is measured by the ratio of the total perimeter of UGS, *P* over their total area *A*. The fragmentation ratio *P*/*A* gives an indication of fragmentation with a higher value if the number of green parcels increases for a given total surface. It is also related to the shape of polygons, with lower values corresponding to a shape closer to a circle and larger values corresponding to elongated shapes.
(iii) The *privatisation index* is measured by the ratio of private (denoted *G* for ‘gardens’) to total UGS cover (*A*), i.e. *G*/*A*.
(iv) The *accessibility index* is measured by the average distance, per neighbourhood, from each cell to the nearest public UGS through the road network. The calculation is unweighted.

**Fig 1:**
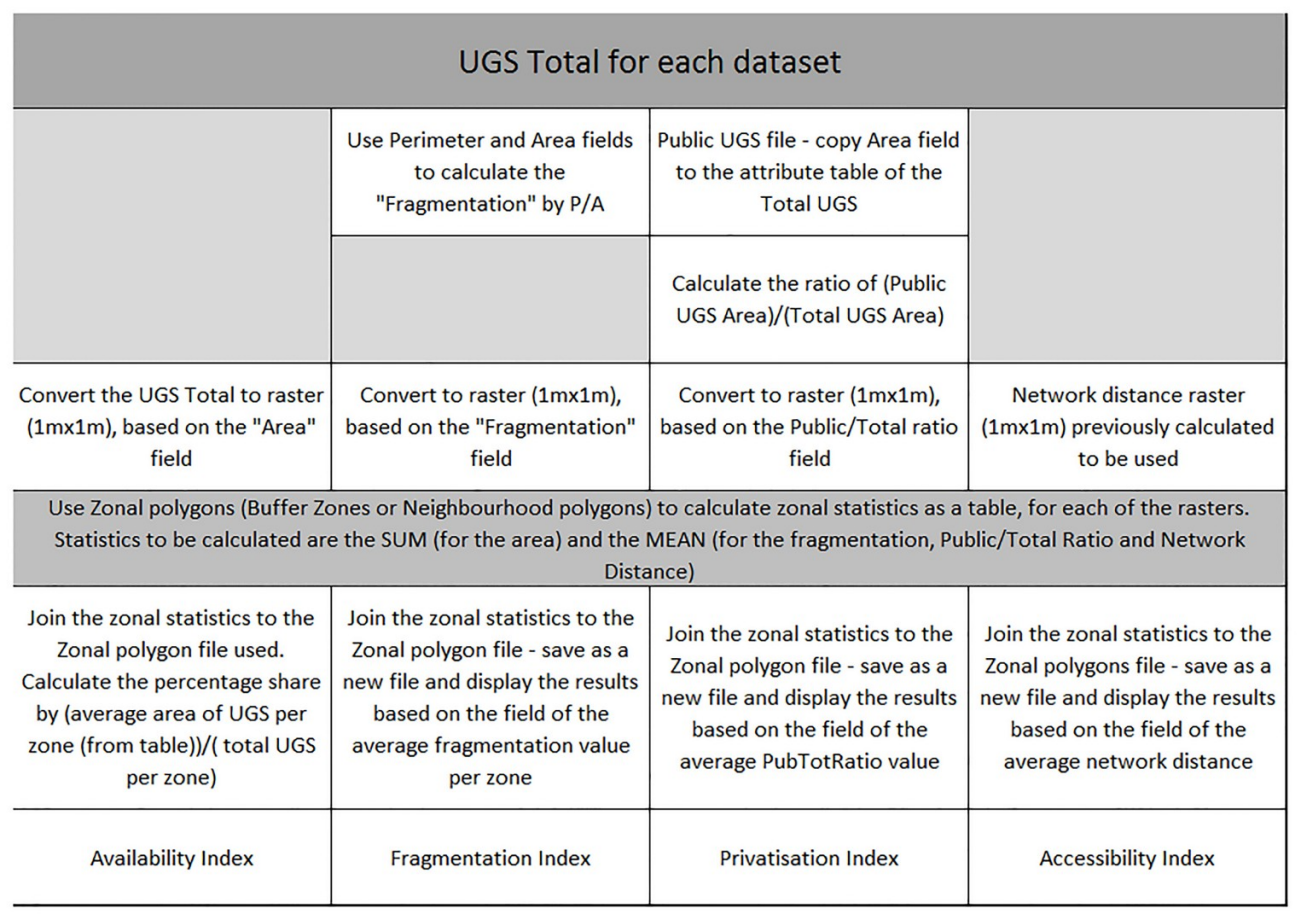
Workflow—Index.

The patterns of UGS distribution are observed through two spatial lenses. First, we account for spatial heterogeneity in UGS provision and access by computing the four indices for a series of distance intervals from the city centre. Centrality is assumed to play a role in the substitution between private and public green as well as in the density of the provision of UGS and therefore its accessibility. Second, we analyse spatial disparities by computing the indices at the neighbourhood administrative levels (Fig 1). The reference surface for the above indicators is therefore alternatively the area corresponding to distance interval rings, the neighbourhood area, or the total regional surface for aggregate average.

For the centrality lens, the location for the region centre was taken as the city hall in Brussels. Buffer rings were then created around this centre at the following distances: 1, 2, 4, 6, 8 and 10 kilometres. For the spatial heterogeneity lens, infra-urban neighbourhoods were defined using 145 statistical entities (so-called “quartiers”) built by a regional statistical body (“Monitoring des Quartiers de la Région de Bruxelles-Capitale”, see https://monitoringdesquartiers.brussels/).

## GIS data and UGS identification

**Fig 2:**
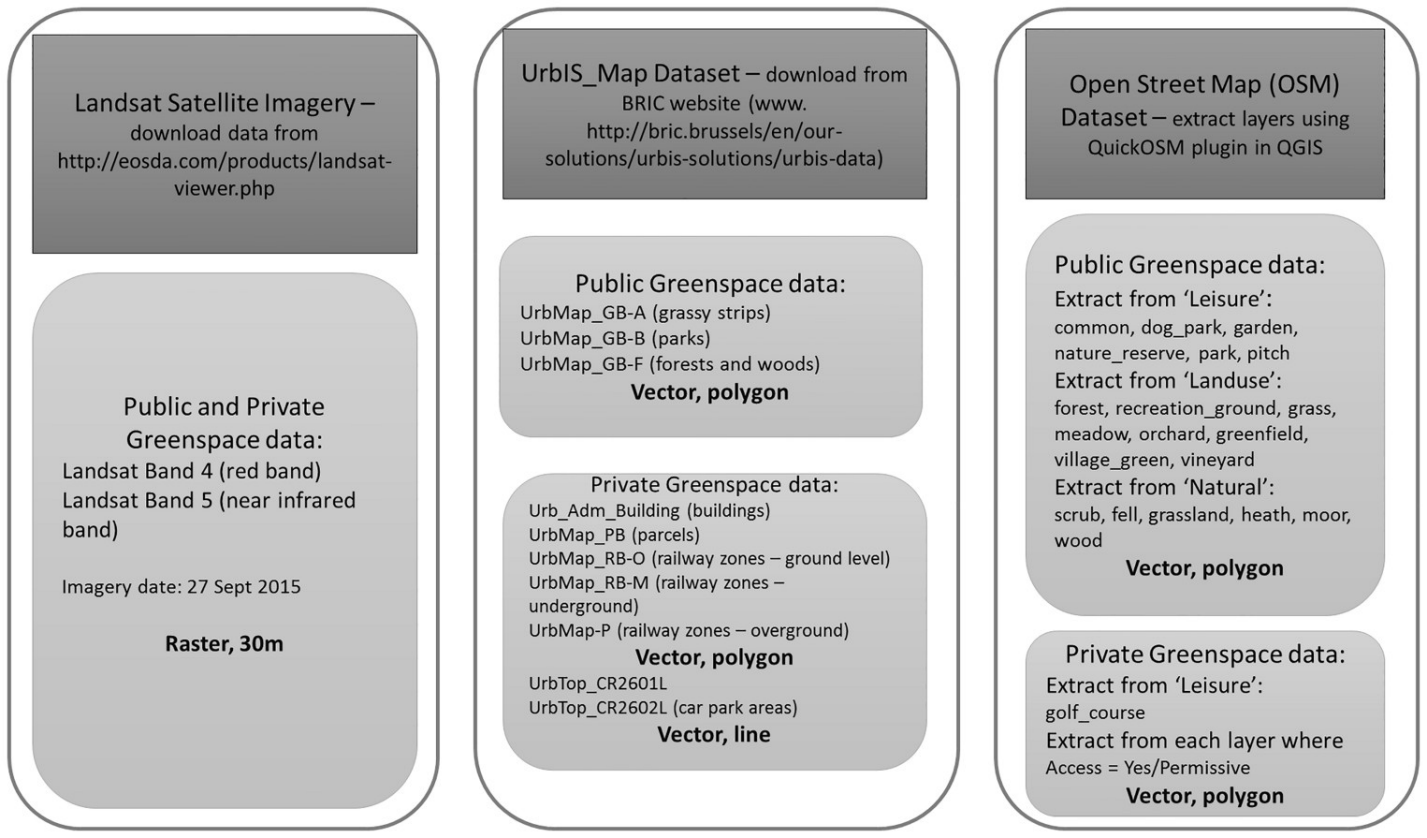
Data downloaded for the three datasets—OSM, UrbIS and Landsat 8.

We identify objects contained in the three databases which could correspond to vegetated areas or which could be used to indirectly extract UGS objects. Resulting UGS objects are therefore likely to differ between datasets (Fig 2) and so the above metrics.

### Identifying UGS with Landsat

The Landsat 8 satellite image covering the Brussels region was downloaded from the Landsat Viewer website (courtesy of the United States Geological Survey—http://lv.eosda.com/). The image used was captured on 20 July 2016, with 0.31% cloud cover, a 56.84° sun elevation angle and a cell size of 30m. Bands 4 (*Red*) and 5 (near infrared, *NIR*) were downloaded, clipped to the area of interest and reprojected from UTM WGS84 to the Belgian 1972 coordinate reference system.

**Fig 3:**
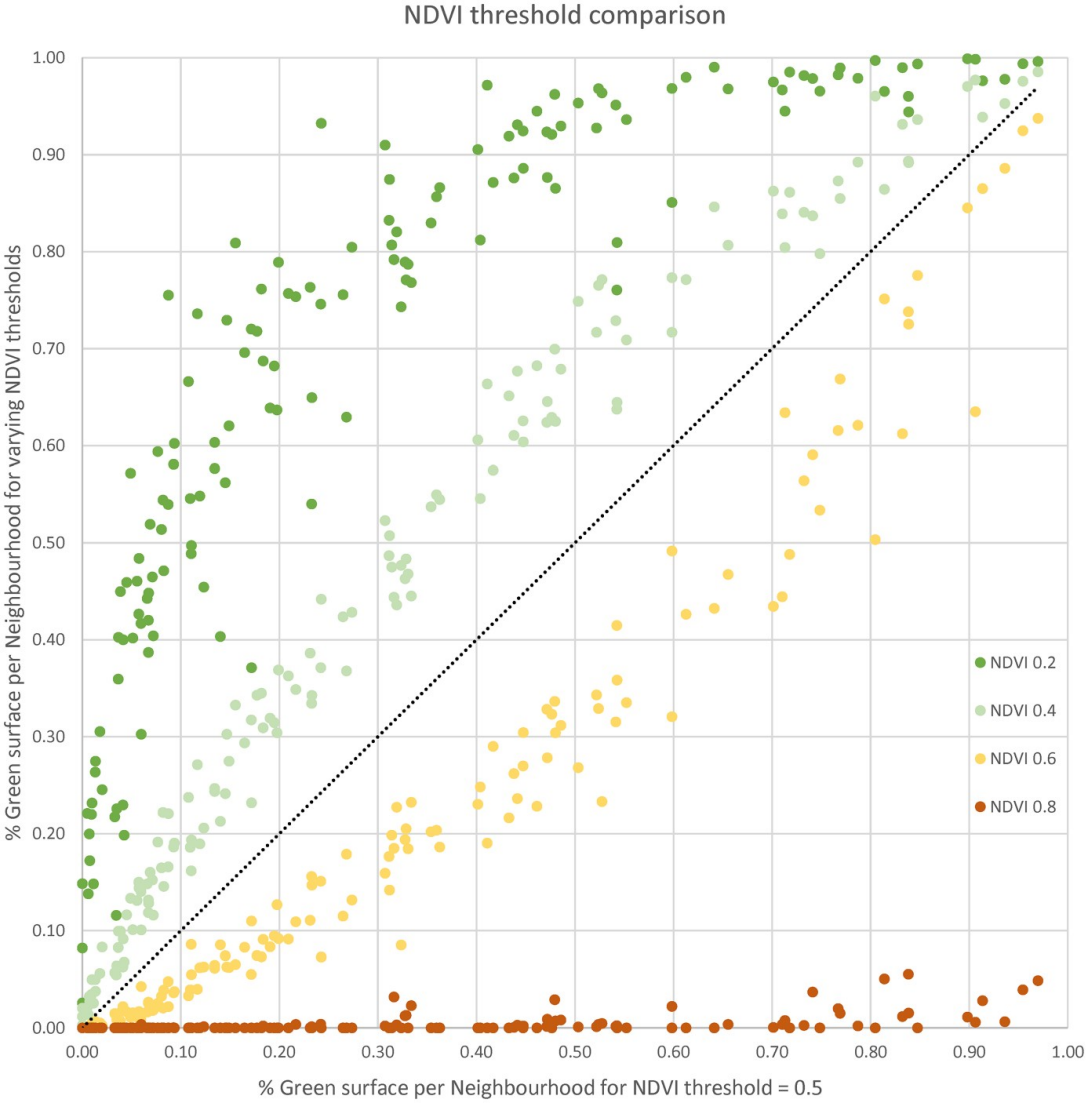
NDVI threshold comparison.

Bands 4 and 5 were corrected for reflectance and sun angle, and the Normalised Difference Vegetation Index (NDVI) calculated, according to the formula (*NIR* − *Red*)/(*NIR* + *Red*). The resultant raster was then reclassified to select only those NDVI values greater than 0.5 (the higher the value the thicker the vegetation) as this value and above represent the most accurate representation of vegetation according to the Land Sciences Team at the United States Geological Survey—https://phenology.cr.usgs.gov/ndvi_foundation.php). This was also confirmed through visual examination. Fig 3 shows a graph comparing the percentage of green represented per neighbourhood using different values of NDVI to our chosen NDVI value of 0.5. It can be seen that values of 0.4 and 0.6 would be consistent with the distribution of that of 0.5, while threshold values above 0.5 and below, definitely change the distribution (and not linearly). We also inspected the spatial pattern of 0.4 and 0.6 against 0.5, which shows that the same spatial structure is preserved, while noise is added in the central streets if thresholds are decreased and many important green features are missed if thresholds are increased. The reclassified raster was then converted to a vector in order to calculate indices for the area covered by the vegetation representing urban green space in the city. This area is the total area covered by UGS in Brussels as it is not possible to distinguish between public and private UGS from this dataset. Compared with UrbIS and OSM, the total green space area from Landsat should in principle represent the upper limit of UGS provision.

### Identifying UGS with UrbIS

Vector shapefiles from the UrbIS Map and UrbIS Admin datasets were downloaded from the BRIC website (http://bric.brussels/en/our-solutions/urbis-solutions/urbis-data). UrbIS Map provides polygons covering the public urban green spaces in the Brussels region, including forests and woods, parks and grassy strips. For the calculation of private UGS in the next steps, parcel data and railway zones from UrbIS Map and buildings and car park areas from UrbIS Admin were used. The data is provided in the Belgian 1972 coordinate reference system.

**Fig 4:**
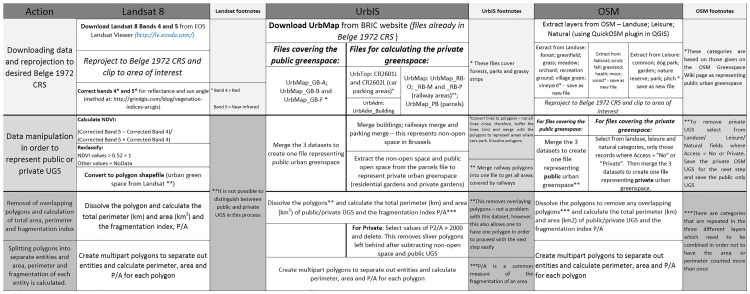
Workflow—OSM, UrbIS and Landsat creation of private and public UGS.

In order to determine the total public UGS as given by the UrbIS dataset, the three relevant files from the UrbMap dataset (UrbMap_GB−A; UrbMap_GB−B and UrbMap_GB−F) (see Fig 4) were merged into one layer. The polygons were then dissolved to remove any overlapping polygons in order to calculate the correct fragmentation index of the public UGS. The polygons were then split into single parts in order to later calculate the fragmentation values (see below) for each UGS entity.

Data for private UGS in Brussels was created by extracting the non-open space (i.e. buildings, railways and car parking areas) from the parcel data. The public UGS were also extracted from the parcel data. The resultant areas represent private UGS, such as residential gardens. After removing the non-open space in this manner, it was necessary to remove sliver polygons that resulted, as they were too small to be considered as private UGS and therefore should not be included in calculations. In order to do this, the fragmentation was calculated for each polygon, and those with a value of greater than 2000 were deleted, as these represent the majority of the sliver polygons that were created through this method. As previously stated, lower fragmentation values imply a more circular shape, while higher values imply long, thin shapes, such as the sliver polygons that were created in this step. The polygons with a value of less than 2000 are then of a shape and size to be considered as a private UGS. The value of 2000 was chosen through manual inspection of polygons with that value or greater.

### Identifying UGS with OSM

Vector data from Open Street Map (http://openstreetmap.org) was extracted using the QuickOSM plugin in QGIS. The “Land use”, “Natural” and “Leisure” categories covering the Brussels area were extracted separately and from each of these files, specific categories of land use, natural and leisure pertaining to urban green space were selected. The following lists the relevant categories. From “Land use”: forest; recreation ground, grass, meadow, orchard, greenfield, village green and vineyard. From “Leisure”: common, dog park, garden; golf course; nature reserve; park and pitch. Finally, from “Natural”: scrub; fell; grassland; heath; moor and wood. These categories were listed on the OSM Wiki as being representative of green space access (http://wiki.openstreetmap.org/wiki/Green_space_access). These files were then merged into one file representing all open green space and reprojected to the Belgian 1972 coordinate reference system.

The layers mentioned above that were selected from “Landuse”, “Leisure” and “Natural” were merged to create a single feature layer representing the public open space. The polygons were dissolved to remove any overlapping polygons, which in this dataset was a necessary step due to overlapping coverage by different categories. A total fragmentation value was calculated at this point. This layer only contained records where the field “access” equalled “yes” or “public” or was left empty, as these represent urban green space with public access. The dissolved polygon was then separated into single parts and the perimeter, area and fragmentation calculated for each entity.

Private OSM data was extracted from the downloaded “Land use”, “Leisure” and “Natural” vector data where the “access” field was listed as ‘no’ or ‘private’, indicating restriction of access to these areas. These polygons were then saved and merged into one file representing the private UGS represented on Open Street Map. The polygons were dissolved to remove any overlaps, and the private fragmentation value calculated. The dissolved polygons were then separated to single parts in order to calculate separate fragmentation values.

Fig 4 sums up the procedure to identify UGS for the three datasets.

## Results

We compare UGS provision and access with the four indices and the three data sets, first as an aggregate for Brussels, second along centrality, and third per neighbourhood.

### Aggregate measures

**Fig 5:**
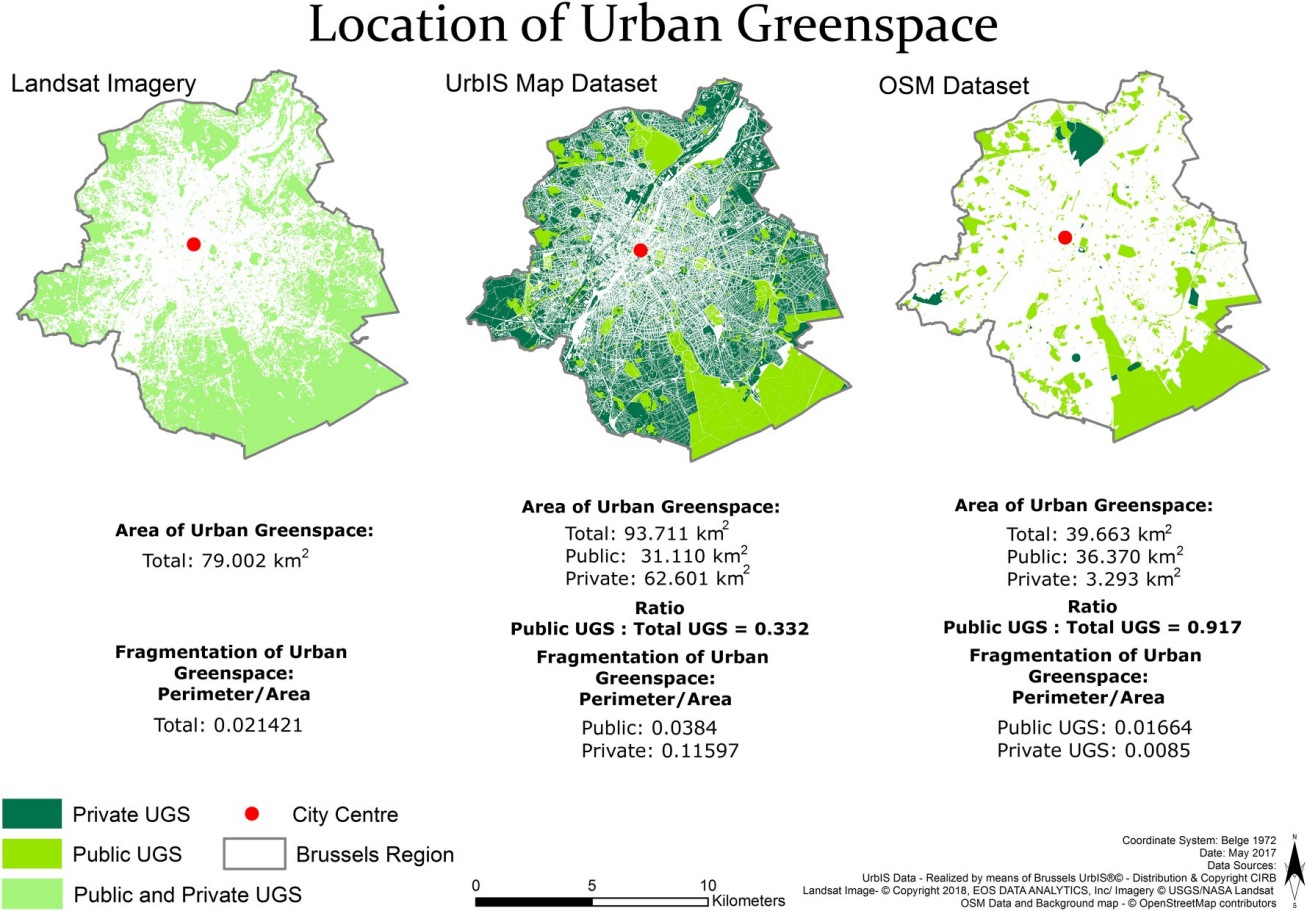
Provision of UGS in Brussels for the 3 datasets—Landsat, UrbIS and OSM.

The provision of UGS is mapped in Fig 5 for each dataset. UGS identified with Landsat cannot be distinguished between public and private and are therefore displayed as a total. Also, the privatisation and accessibility (to public green) cannot be computed with Landsat.

UrbIS and Landsat are comparable in terms of total availability of UGS. It is noticeable that about half of the surface of a city like Brussels is actually green, which is important in absolute terms and challenges the idea that there is a clear cut line that defines a city as a non natural system, as opposed to leafy suburbs. Landsat surprisingly provides a slightly lower value of total availability and not the upper limit assumed earlier. This highlights the fact that interior private parcels in cadaster-based data (UrbIS) cannot be assumed to be green, nor to provide green benefits to owners and neighbours. This said, we find with UrbIS that there is about twice as much private green as public green. This is reflected also through the privatisation index in UrBIS (0.67). The interior of private parcels is very important to the overall greenness of a city. Hence, much of the policy that would aim at increasing green space benefits is actually not in the hands of public decision makers even within a city. This is very important in light of substitution effects e.g. stressed by [12, 18]. The method used to calculate the private urban green space with UrbIS data does tend to overestimate the green due to the point mentioned above that not all interior private parcels are green—this method could be improved through combining the results of this method, with the results of the Landsat green extraction to get a more accurate value. However, as we are comparing datasets here, this was not done in this instance.

We see that OSM is unable to fully capture total greenness in the case of Brussels. The availability index based on OSM is about half the availability in UrbIS or Landsat. This difference comes mostly from the fact that OSM does not adequately account for private UGS. The privatisation index is then meaningless in OSM. OSM provides similar locations for public green as UrbIS, and these public spaces are mostly vegetated as reflected by Landsat. Exceptions are found for some UGS (e.g. around Laeken castle, Anderlecht golf course) that the OSM contributors depicted as private because of restricted (fee) access. Those exceptions do not affect the indicator of overall accessibility to public green, i.e. 35m average distance with UrbIS and 33m with OSM, which is very low and hints at a good distribution of public green in Brussels. In that regard, swapping between the two datasets is not affecting our understanding of access to green space, which is good news for replication and comparative purposes given the ubiquity of OSM.

Fragmentation is the aggregate indicator whose behaviour across datasets is the most difficult to relate. Total fragmentation is similar for Landsat and OSM while we know the latter does not account for private space, indicating that both private and public space would have similar fragmentation. This however is not supported by the different measures obtained for public and private space in the UrbIS data. Further, the higher *P*/*A* ratio for public space is definitely unexpected. These aggregate results cast serious doubts on any fragmentation study that would compare cities based on different data sources. We believe only the spatial heterogeneity (see below) within a single urban area and for a single dataset can make sense.

### Centrality effects

**Fig 6:**
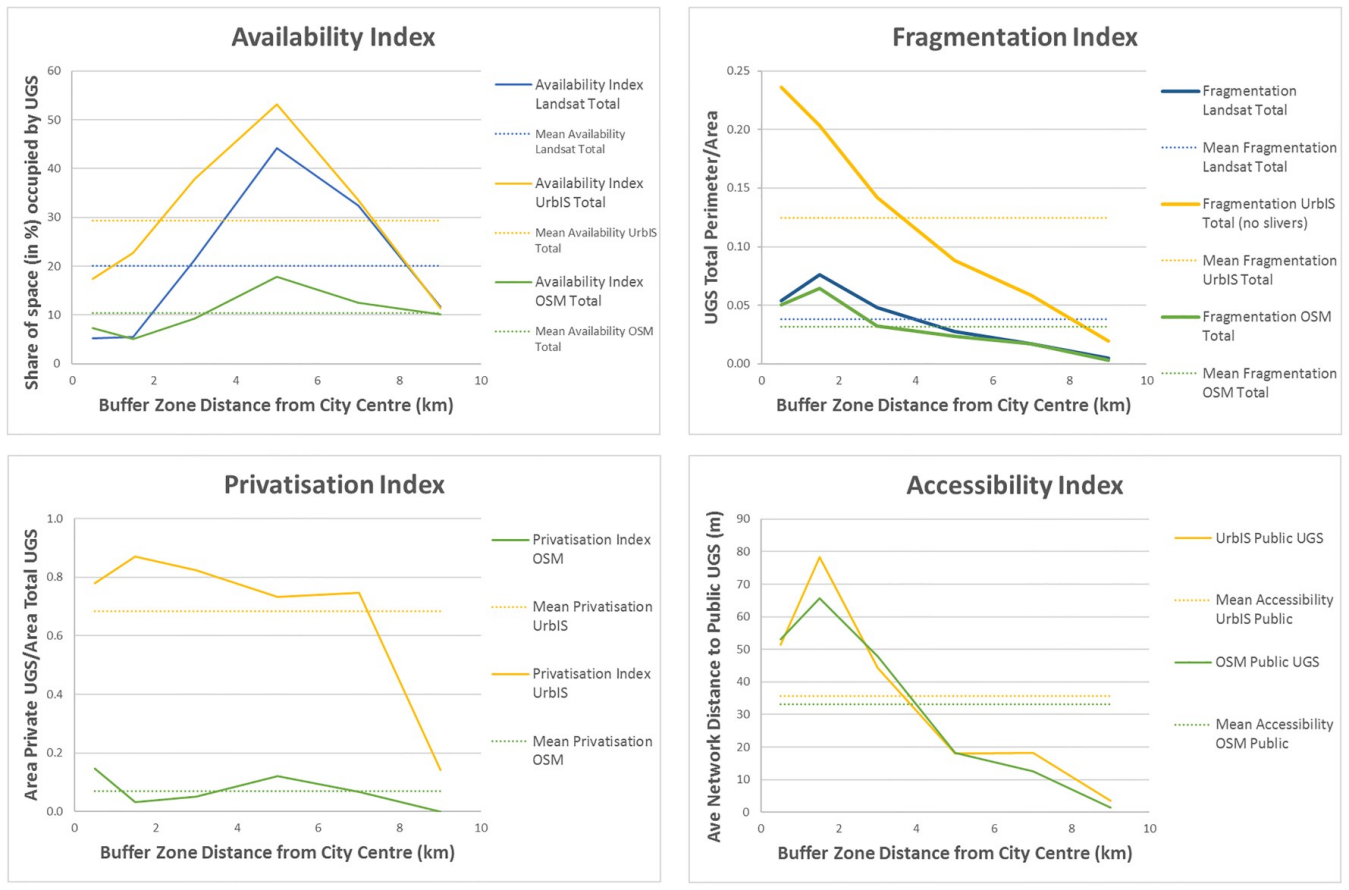
UGS provision and access indicators per distance bands (average).

We now turn to analysing indices across distance profiles (Fig 6).

With regards to the availability index, we find that all datasets show that the total provision of green space increases with distance to the city centre until around 6km before it starts decreasing towards the limit of the city. This profile is less pronounced for OSM data, but is still evident with the same distance for the maximum. This agreement for profiles between datasets is quite remarkable, especially given the many differences in UGS type represented by each dataset.

The absolute values of availability are quite different across datasets, meaning again that comparing values from different data sources would not be robust in this case. UrbIS and Landsat typically show higher total availability than OSM as they take into account private UGS. The availability values converge at the outskirt of the city where the weight of a large public green space (Bois de la Cambre located in the South) takes up most of the green surface while the reference rings are smaller in surface because of border effects. UrbIS and Landsat differ mostly in availability when approaching the centre, showing that assuming that private courtyards are green is an assumption that holds better in suburbs than close to the centre.

We should expect an inverted curve for the distance to UGS, i.e. accessibility. Given that the maximum of availability is near 6km, we can expect minimum distance to access UGS along network at the same distance. This is not what we find however: the accessibility is high (distance is low) at 6km but even more at further distances. Despite a maximum access distance around 2 kilometres from the city centre in the two datasets, the general profile is downward sloping. This follows the fact that the core area lacks private green, which is accounted in the availability but is not in the accessibility index. It is important to note here that data sources make no difference in interpretation, but to realize that the availability of UGS and accessibility to public UGS dimensions are not a direct complement in light of a distance gradient.

Fragmentation shows downward sloping profiles with distance for all datasets, though far more clearly with the UrbIS data. This decreasing trend may be surprising if you think of the presence of more gardens in the suburbs. Yet, plots are smaller to the center and in addition there is a provision of larger and more cohesive public UGS towards the periphery. As for the accessibility index, we see in OSM and Landsat a small increase of fragmentation between the centre and a distance equal to 2km, which does not appear in UrbIS. As mentioned above in the aggregate view, absolute fragmentation values are difficult to compare across datasets. There is only a convergence of values at the urban fringe.

Privatisation absolute values are similarly affected by data sources. There is a rejoinder in values between UrbIS and OSM again because of the large forested area at the very periphery of study area. This coincidence of border effects and public green excepted, we see that the measure of UGS privatisation for a single dataset is not much affected by distance. It is an interesting finding that there is no substitution or compensation possible between private and public green space and therefore that the provision of public green within an urban area is to the advantage of its most remote inhabitants. Our index is one of privatisation of UGS, not one of ‘private garden’ availability. There is then an imbalance between the two datasets with an overestimation in UrbIS and an underestimation in OSM due to contributors not capturing private green as extensively as public green. These imbalances appear clearly when comparing with green space as shown in Landsat, however, as this study is a comparison, datasets have not been combined in order to give a more accurate output regarding the presence of private green space.

### Neighbourhood differences

**Fig 7:**
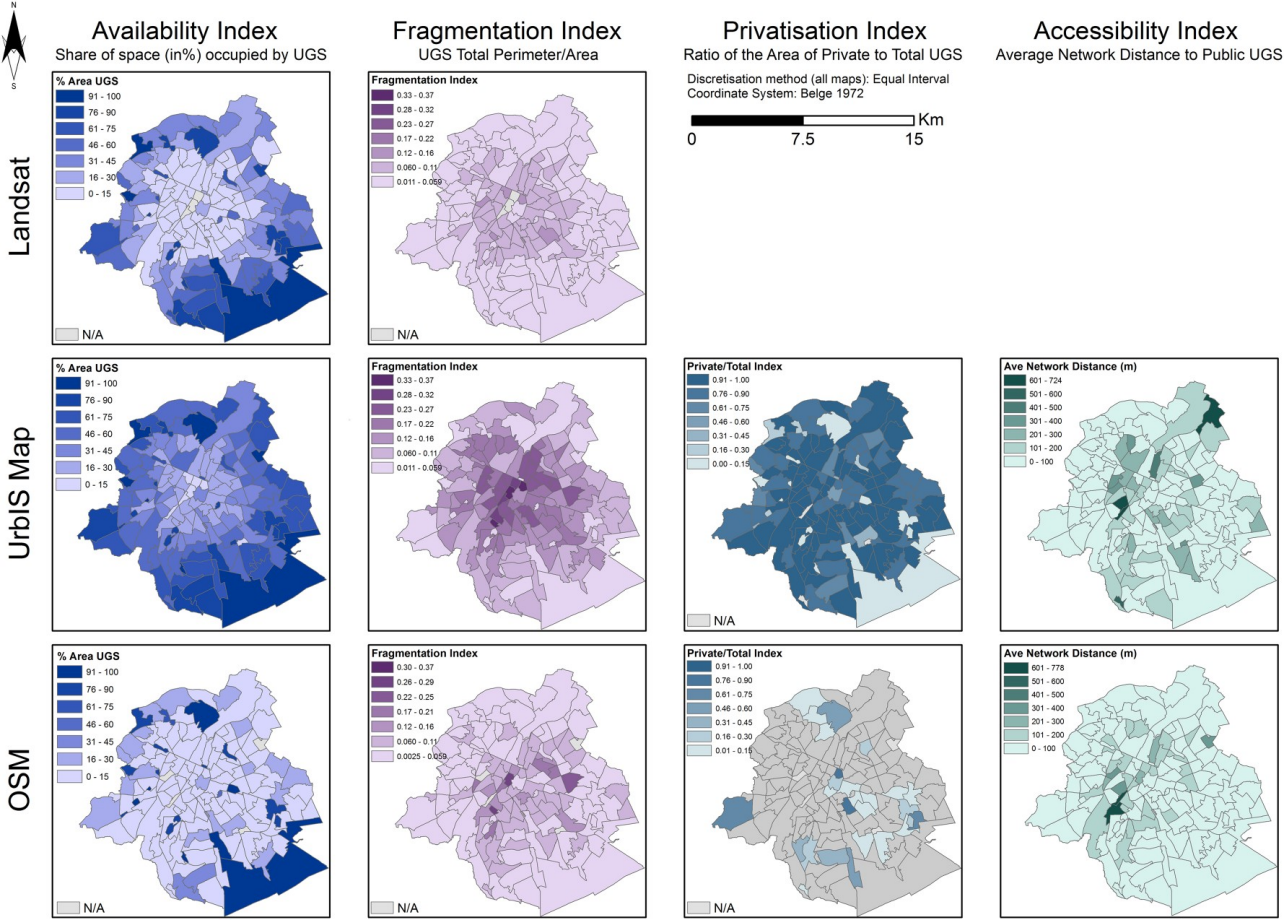
Comparison of average indicators for total UGS per neighbourhood (quartier), per dataset. **Discretization method: quantile**. We use a quantile-based discretization method to stress the ordered differences between neighbourhoods. A mapping of the indicators using equal intervals is proposed in the Supporting information section to ease the comparison between cities (S1 Fig).

We now analyze the spatial distribution of each indicator and seek to identify spatial heterogeneities that would not be related to centrality. The maps shown on Fig 7 are based on a quantile discretisation in order to allow for row-wise comparisons, i.e. comparing the relative spatial distribution across indices rather than across data.

We see more clearly now that the availability and fragmentation indictors are inverted maps, irrespective of the datasets. We see also that most of the spatial differentiation is linked to centrality: there is lower provision / stronger fragmentation of UGS in central neighbourhoods and higher provision / lower fragmentation in outer neighbourhoods.

Conversely to availability and fragmentation, the privatisation index does not undergo a clear centrality effect, as shown on Fig 6. There is no other particularly strong spatial structure or any cluster of high or low privatisation. This is the case for both UrbIS and OSM. What is more striking is that the interpretation one can make from the two maps would almost be reversed. Changing the dataset definitely impacts the understanding of UGS privatisation and its spatial disparities, yet independently of centrality in both cases.

With regards to accessibility, i.e. network distance, there is some centrality effect but also an additional structuring along a South-West/North-East axis. This is a known urbanisation structure effect in Brussels due to the Senne canal. Crossing the canal leads to additional travel time to the nearest public UGS. This effect adds-up to centrality effects present in UGS availability. The structure seems robust to changing the dataset from UrbIS to OSM.

## Conclusion

We have analysed how four main dimensions of UGS provision and access vary across different datasets due to heterogeneity in both the types of objects considered and minimum map units. Overall our analysis shows that it is important to control for different data sources as soon as the absolute values of the indicators are important, hence for comparative purposes. Sources do not so much impact relative values and the spatial pattern of indicators especially in terms of centrality. In this sense and in the case of Brussels our analysis supports the robustness to data change of our indicators and [17]’s indicators of green access. When the nature of the urban green however must be part of the assessment, particularly when there is a need to differentiate private and public green space, then data sources is again an important concern.

Landsat data cannot distinguish private from public UGS, while OSM data does not depict private UGS at all. Retrieving private green space from cadasters remains difficult, which we only see because we have also used an imagery and NDVI. The fact that we systematically analysed different dimensions has been instrumental to finding out how the private/public UGS impact our understanding of access and provision. This calls for continued application of multi-dimensional approaches in the future because it not only enlightens important facets of UGS provision, but also because methodologically it helps with understanding differences between indicators across datasets.

When the public/private nature of reported UGS is controlled for, there are still important differences in absolute values of each indicators across datasets. This certainly challenges the possible comparison of aggregate values for different cities having different data sources, in particular different ways to implement local cadasters or comparison that would mix raster imageries on one side and vector products on the other (volunteered product or institutional ones). However we find that the interpretation of intra-urban spatial variations are not much affected by such changes in data source beyond this private/public green space effect. More specifically, we find that radial gradients, i.e. how provision and access dimensions change with centrality, are robust to data effects. This assessment is particularly important because distance relates to residential choice components, the endogeneity of green space in that choice and potential substitution of private and public UGS.

Our results that swapping datasets is not necessarily impacting interpretation is a rejoinder to [42] who conclude that despite differences in UGS location derived from CORINE land cover and Ordnance Survey master map, a similar relationship between UGS access and mortality can be found. Our results are also in line with [18] who found that informal UGS were more sensitive to data selection than formal UGS. We support this idea of unequal sensitivity to data for different kinds of green space, especially public/private and smaller green bits. Compared to those studies, we stress that a radial (centrality) perspective makes interpretation stronger. Centrality is a strong determinant of the relative value of availability, fragmentation, and accessibility. The latter we suggest is also affected by network shapes and exogenous features (rivers).

Finally, we find that OSM dataset tends to resemble the official cadaster-based approaches (such as UrbIS in Brussels) quite well, but more so closer to the city centre. This is in line with [48] showing that the concentration of OSM contributions in more densely populated areas may have an impact on data validity. In present times, using voluntary geographic information to analyse UGS provision and access, and maybe social media for UGS use, necessitates taking into account this spatial heterogeneity in data completeness and reliability. Future research should aim at a comparison of case studies to allow for generalizing the research findings. Indeed, differences in data quality may lead to variations in the results.

## Supporting information

S1 Fig Comparison of average indicators for total UGS per neighbourhood (quartier), per dataset. Discretisation method: equal intervals.
(EPS)
